# Kinetics of ventilation-induced changes in diaphragmatic metabolism by bilateral phrenic pacing in a piglet model

**DOI:** 10.1038/srep35725

**Published:** 2016-10-19

**Authors:** Thomas Breuer, Nima Hatam, Benjamin Grabiger, Gernot Marx, Bradley J. Behnke, Joachim Weis, Ruedger Kopp, Ghislaine Gayan-Ramirez, Norbert Zoremba, Christian S. Bruells

**Affiliations:** 1 Department of Anaesthesiology, University Hospital of the RWTH Aachen, Aachen, Germany; 2 Department of Intensive and Intermediate Care, University Hospital of the RWTH Aachen, Aachen, Germany; 3 Department of Thoracic and Cardiovascular Surgery, University Hospital of the RWTH Aachen, Aachen, Germany; 4 Department of Kinesiology, Johnson Cancer Research Institute, Kansas State University, Manhattan, Kansas, USA; 5 Institute of Neuropathology, University Hospital of the RWTH Aachen, Aachen, Germany; 6 Laboratory of Pneumology, Katholieke Universiteit Leuven, Leuven, Belgium; 7 Department of Anaesthesiology, Sankt Elisabeth Hospital, Gütersloh, Germany

## Abstract

Perioperative necessity of deep sedation is inevitably associated with diaphragmatic inactivation. This study investigated 1) the feasibility of a new phrenic nerve stimulation method allowing early diaphragmatic activation even in deep sedation and, 2) metabolic changes within the diaphragm during mechanical ventilation compared to artificial activity. 12 piglets were separated into 2 groups. One group was mechanically ventilated for 12 hrs (CMV) and in the second group both phrenic nerves were stimulated via pacer wires inserted near the phrenic nerves to mimic spontaneous breathing (STIM). Lactate, pyruvate and glucose levels were measured continuously using microdialysis. Oxygen delivery and blood gases were measured during both conditions. Diaphragmatic stimulation generated sufficient tidal volumes in all STIM animals. Diaphragm lactate release increased in CMV transiently whereas in STIM lactate dropped during this same time point (2.6 vs. 0.9 mmol L^−1^ after 5:20 hrs; p < 0.001). CMV increased diaphragmatic pyruvate (40 vs. 146 μmol L^−1^ after 5:20 hrs between CMV and STIM; p < 0.0001), but not the lactate/pyruvate ratio. Diaphragmatic stimulation via regular electrodes is feasible to generate sufficient ventilation, even in deep sedation. Mechanical ventilation alters the metabolic state of the diaphragm, which might be one pathophysiologic origin of ventilator-induced diaphragmatic dysfunction. Occurrence of hypoxia was unlikely.

Continuous mechanical ventilation (CMV) in acute respiratory insufficiency is associated with diaphragmatic inactivity, leading to atrophy and loss of diaphragmatic force. These detrimental changes in the diaphragm increase morbidity and mortality of intensive care patients. Unlike locomotory skeletal muscles, which develop atrophy after several days of inactivity^1^, atrophy in the diaphragmatic muscle and the loss of its functionality begin within hours after the onset of CMV^2^,^3^. This rapid process of diaphragm dysfunction with CMV is commonly referred to as ventilator-induced diaphragmatic dysfunction (VIDD)^4^,^5^. The mechanisms responsible for VIDD are multifactorial, although the process of diaphragm dysfunction with disuse is fundamentally linked to production of reactive oxygen species (ROS)^6^. Another potential mechanism contributing to the onset of VIDD is an imbalance in diaphragmatic oxygen and nutritive supply-demand. Specifically, the time-dependent reduction in diaphragm blood flow after the onset of CMV^7^, potentially causing ischemia, associated tissue hypoxia and a shift to anaerobic metabolism promoting muscular fatigue are suspected mechanisms^7^,^8^. Indeed, hypoxia-inducible factor 1-alpha (HIF1-A) mRNA was up-regulated with CMV in the diaphragm, although there were no elevations of other hypoxia sensitive markers (e.g., vascular endothelial growth factor)^9^. Therefore, whether diaphragm tissue hypoxia associated with prolonged inactivation is induced and may contribute to VIDD is unknown.

Several studies indicated that maintaining a degree of activity in the diaphragm during MV through spontaneous breathing^10^,^11^ or pressure support ventilation^12^ mitigates diaphragmatic function and/or atrophy. Furthermore, patients’ capability to activate the diaphragm during MV needs to be assessed, which can be contraindicated by a variety of comorbidities. The ideal paradigm to mitigate VIDD would be an immediate start of ventilator-adapted spontaneous breathing after endotracheal intubation^13^. Conversely, stimulation of the diaphragm through artificial activation of both phrenic nerves during deep sedation and/or in those patients lacking central neuromuscular drive could be a suitable technique to implement spontaneous breathing without the high-level attentiveness required to activate the diaphragm^14^.

Based on these clinical and scientific questions, this study was conducted under two objectives: Firstly, we examined the feasibility of phrenic nerve stimulation of conventionally epimyocardial applied pacer wires to activate the diaphragm despite deep sedation. Secondly, we sought to investigate whether artificial activation would preserve aerobic metabolism within in the diaphragm whereas CMV would not, at least during the period of investigation. Changes in pyruvate/lactate ratio were used to address the latter as this ratio is indicative of changes in oxygenation state and resultant shifts in metabolism in several tissues, including brain and muscle^15^,^16^,^17^.

Measures were made in the diaphragm during 12 hrs of stimulated or mechanically-ventilated sedated piglets. In addition to metabolic measures, voltage outputs were recorded to test the model for its clinical relevance.

## Methods

The study was approved by the appropriate governmental institution (Landesamt für Natur-, Umwelt- und Verbraucherschutz, LANUV NRW, Germany, reference number: AZ 84-02.04.2012.A224) and conducted in accordance with the principles for care and use of animals based on the Helsinki Declaration.

### Ventilation and anaesthetic model

Healthy, 40–50 kg, female German landrace piglets were separated into 2 groups (each n = 6). The first group was mechanically ventilated (CMV) and the second group breathed via phrenic nerve stimulation to mimic spontaneous breathing (STIM). Both groups received intravenous anaesthesia with pentobarbital and fentanyl to induce a deep sedation level of anaesthesia.

In both groups premedication was given intramuscularly with a mixture of 1 ml 1% atropine sulfate and 0.2 ml kg^−1^ bodyweight^−1^ azaperone. Prior to peripheral venous access, intramuscular sedation was initiated with 1 mg ketamine kg^−1^ bodyweight^−1^. Thereafter, anaesthesia was induced by an intravenous injection of pentobarbital (10–20 mg kg^−1^ bodyweight^−1^) and afterwards maintained via continuous intravenous infusions of pentobarbital (5–15 mg kg^−1 ^h^−2^) and fentanyl (0.02 mg kg^−1 ^h^−2^). All animals were orally intubated and connected to a ventilator (Evita4, Draeger, Luebeck, Germany). Continuous intravenous fluid (Saline 0.9%; 10 ml kg^−1 ^h^−2^) was supplied to ensure normovolemia and diuresis was monitored via a transurethral catheter.

Initially all animals were mechanically ventilated with pressure-controlled ventilation until all surgical procedures were completed to ensure normocapnia (end-tidal CO_2_ (etCO_2_) 35–45 mmHg with a tidal volume of 6–8 ml kg^−1^ bodyweight^−1^). Body temperature was maintained at 38 °C via a circulating heated water pad. Both groups received an initial infusion of 500 mg of cefuroxime. In both groups, a median thoracotomy was performed and stimulation electrodes (TME, Osypka, Rheinfelden, Germany) were stitched adjacent to the phrenic nerves bilaterally. Two microdialysis probes (CMA 20, CMA Microdialysis, Kista, Sweden) were inserted into the costal diaphragm. After insertion of bilateral drainages the thorax was closed.

After this surgical procedure the animals were randomized into two groups. One group achieved pressure-controlled mechanical ventilation for 12 hrs (CMV, n = 6 animals) with inspiratory pressures resulting in tidal volumes of 6–8 ml kg^−1^ bodyweight^−1^, an I:E ratio of 1:1 and using a PEEP of 5 cm H_2_O (See Supplemental Figure 2). In the second group (STIM, n = 6 animals) this initial pressure-controlled mechanical ventilation was stopped after the surgical procedure and stimulated spontaneous breathing was initiated by bilateral phrenic nerve stimulation as described below with a tidal volume of 6–8 ml kg^−1^ bodyweight^−1^ (See Supplemental Figure 2 for additional information to ventilator settings). Arterial blood gases were taken every 4 hrs for determination of serum lactate and glucose concentrations (See Supplemental Figure 3 for further parameters of arterial blood gas analyses). During the entire experiment ECG activity, blood pressure, central venous pressure and oxygen saturation were recorded continuously. Cardiac output was continuously monitored via thermodilution (PiCCO, Pulsion Medical Systems, Feldkirchen, Germany). From this time point diaphragmatic microdialysis samples were taken continuously in both groups (described below).

After 12 hrs diaphragmatic tissue samples were taken and the animal was euthanized using an overdose of pentobarbital.

### Baseline conditions

Baseline was defined as the time point when diaphragmatic microdialysis was established in all animals after closure of the thorax (4 hrs after induction of CMV). Animals were then divided into the interventional groups.

### Phrenic nerve stimulation technique

In the stimulation group the diaphragm was paced with a Grass stimulator (S48 Square Pulse Stimulator, Natus Neurology Inc, Warwick, RI, USA) using a train rate fixed at 0.5 trains per second with a duration of 0.8 seconds and a minimal delay of 0.01 milliseconds at 20 Hz. Voltage was adapted to maintain tidal volumes (6–8 ml kg^−1^ bodyweight^−1^) and varied from 2 to 12 volts to ensure adequate ventilation with normocapnia (S48 Square Pulse Stimulator, Natus Neurology Inc, Warwick, RI, USA). The animal efforts were supported using automatic tube compensation (ATC, Draeger, Lübeck, Germany) to avoid negative pressure lung oedema.

### Microdialysis measurements of aerobic and anaerobic energy substrates

The microdialysis probe consists of an integrated semipermeable membrane to measure extracellular levels of lactate, pyruvate and glucose (CMA 20, CMA Microdialysis, Kista, Sweden), with an outer diameter of 0.5 mm and a molecular cut-off at 20.000 Daltons. The microdialysis catheter was connected to a precision infusion pump (CMA 102, CMA Microdialysis, Sweden) and continuously perfused with a dialysate containing 147 mmol L^−1^ Na^+^, 4.0 mmol L^−1 ^K^+^, 2.3 mmol L^−1^ Ca^2+^ and 155.6 mmol L^−1^ Cl^−^ (Perfusion fluid T1, CMA Microdialysis, Sweden) at a flow rate of 2 ml min^−1^.

After an initial equilibration time of 60 minutes, microdialysate samples were collected at 20 minute intervals throughout the experiment and immediately stored at −80 °C. Dialysates were analysed with high-pressure liquid chromatography (HPLC) technique (CMA 600 Microdialysis Analyser, CMA Microdialysis, Kista, Sweden) for quantitation of lactate, pyruvate and glucose.

#### Histological measurements

Costal diaphragm tissue was embedded and frozen in liquid butane for histological assessment of muscle fibre dimensions by ATPase assay as described before^18^. Fibre cross-sectional areas were examined using immunofluorescence microscopy (approximately 200 fibres per animal) and analysed using the software ImageJ (v1.46 k; National Institute of Health, Bethesda, MA)^19^.

#### Biochemical measurements

Determination of total protein concentration was assessed using the Bradford method^20^. Proteins were separated by 10% SDS-Page and transferred onto a polyvinylidene fluoride (PVDF) membrane (Bio-Rad Laboratories, Hercules, CA, USA). Ponceau S staining was used to ensure equal loading and proper transfer of the proteins. Western Blots were incubated overnight at 4 °C with appropriate primary antibodies (4-HNE: #AB-46545, Abcam, Cambridge, UK; Calpain-1: #SC-13990, Santa Cruz, Dallas, Texas, USA) and subsequently with the suitable horseradish-peroxidase conjugated secondary antibody (anti-rabbit, #7074, Cell-signalling Technology, Danvers, MA, USA). Proteins were visualized by enhanced chemiluminescence using peroxidase substrate (Clarity Western ECL Blotting Substrate) and analysed using the ChemiDoc System with the Image Lab Software (Bio-Rad Laboratories, Hercules, CA, USA) and normalized to Vinculin (#V9131, Sigma-Aldrich, St. Louis, MO, USA) as a loading control.

### Statistical analysis

A sample size of 6 animals per group was chosen in this pilot study setup. Data were analysed using non-parametric tests. For physiological data the Kruskal-Wallis test followed by a Dunn’s post hoc test was performed. Microdialysis data were analysed in a two-step model: Mann-Whitney-U test was calculated between the two groups to identify interactions between both groups. In-between each group Kruskal Wallis test was chosen to assess the changes during the experiment. Western blot measurements were analysed by a two-tailed t-test. Values are displayed as means ± standard error. In all cases, a level of P < 0.05 was considered as statistically significant. SPSS (Armonk, United States) was used.

## Results

### Clinical data

**Figure 1:**
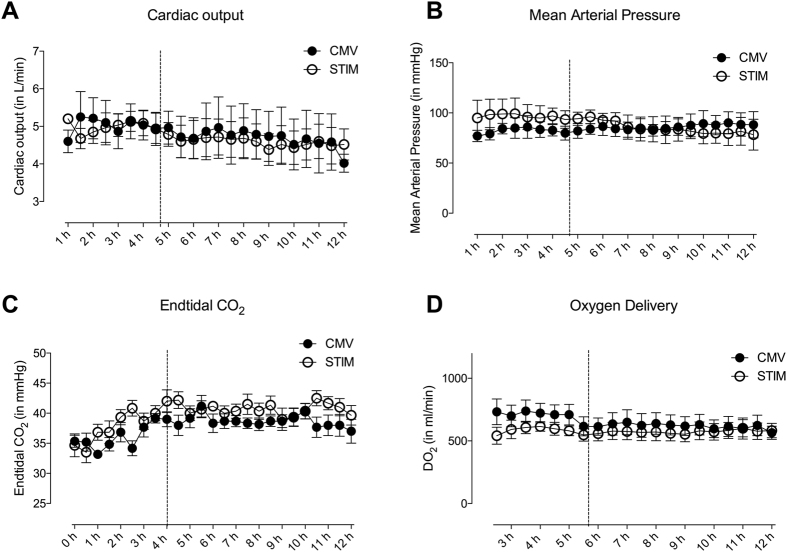
Measured time course of cardiac output (**A**), mean arterial pressure (**B**), entidal CO_2_ (**C**) and oxygen delivery (**D**) in CMV (closed circles) and STIM (solid circles) animals. Broken line displays time point of microdialysis-start where animals were randomized into the interventional groups. Values are displayed as means ± standard error.

Cardiac output was stable and comparable in both groups (CMV: 4.8 ± 1.3 L min^−1^; STIM: 4.7 ± 0.9 L min^−1^) (Fig. 1 panel A). Mean arterial blood pressure did not differ between CMV and STIM during the whole experiment and remained in a normal range (CMV: 85 ± 4.1 mmHg; STIM 89 ± 8.1 mmHg) (Fig. 1 panel B). Central venous pressure did not differ between STIM and CMV and remained in normal ranges. There were no abnormalities in ECG recorded during the investigation.

Normocapnia was maintained in both groups (CMV: 37.2 ± 3.4 mmHg; STIM: 39.4 ± 4.5 mmHg) (Fig. 1 panel C) and oxygen delivery (QO_2;_= Hb × 1,39 × SaO_2_ × 10 × Cardiac output) was comparably stable in all animals (CMV: 651.7 ± 174.7 mL min^−1^; STIM: 588.1 ± 120.6 mL min^−1^) (Fig. 1 panel D).

### Diaphragmatic stimulation

**Figure 2:**
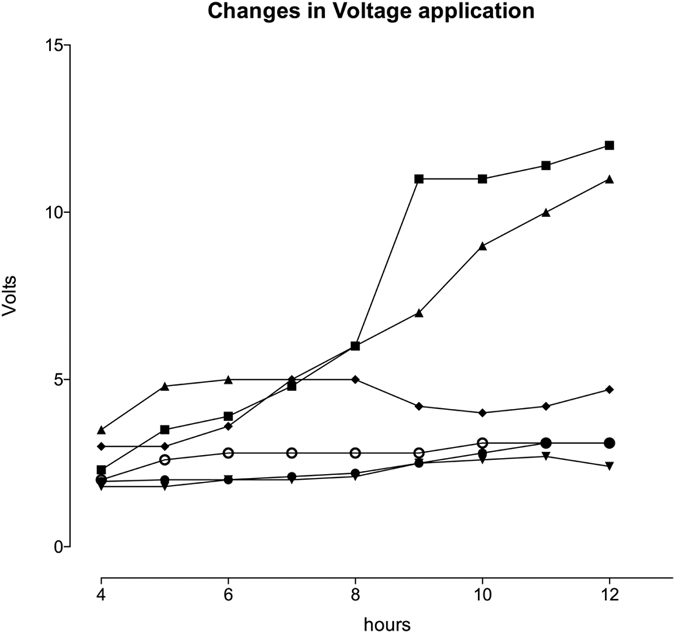
Voltages used to stimulate the phrenic nerves in each stimulated animal during the STIM period. Values are displayed as volts for each animal.

The implantation of the electrodes was successful in all animals, placing the wires in the perineural region around 3 cm above the diaphragm. This yielded better results and required a lower voltage versus placing these wires directly inside the muscle, even when juxtaposed to the nerve. Diaphragmatic stimulation generated sufficient tidal volumes of 6–8 ml kg^−1^ bodyweight^−1^ in all animals of the STIM group. The voltage needed to generate sufficient tidal volumes remained stable in 4 animals, whereas in 2 animals it increased over time (see Fig. 2).

Removing the electrodes via the closed surface wound occurred without complications or indices of nerve damage. Histologic examination of the insertion area did not reveal any signs of neural tissue necrosis or burns (see Supplemental Figure 1).

### Diaphragmatic lactate production during CMV and STIM

**Figure 3:**
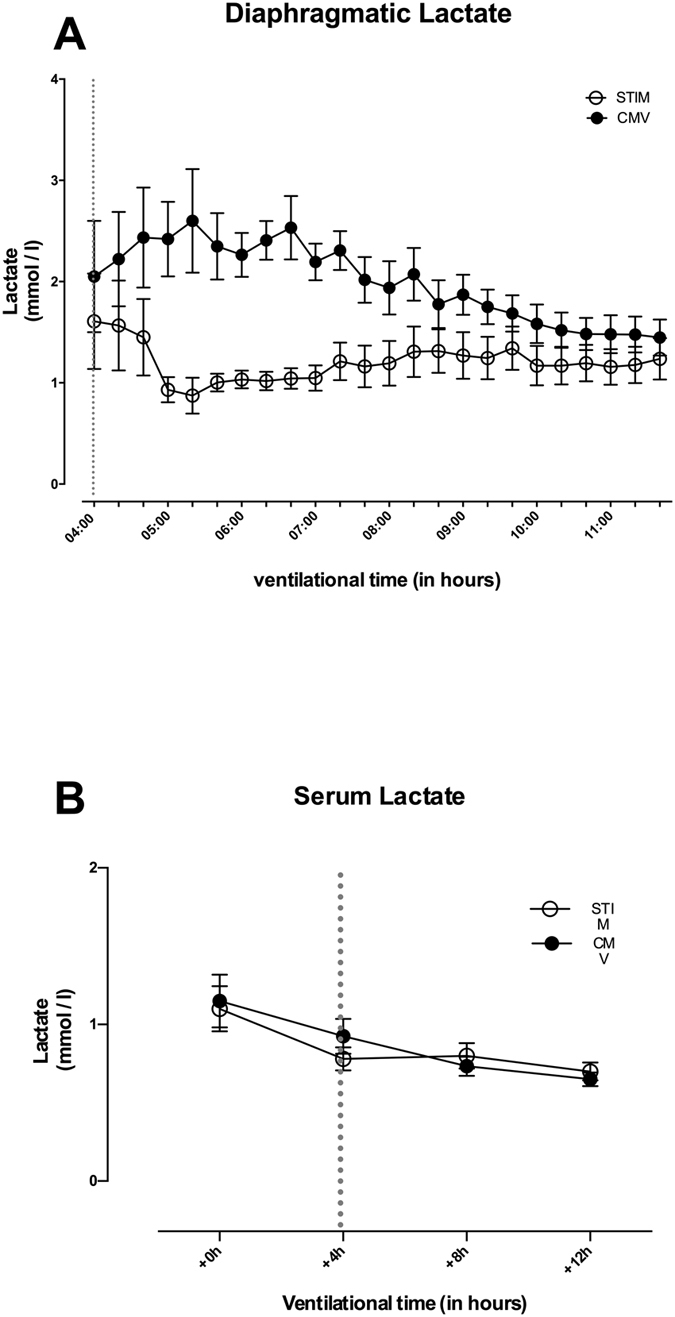
Lactate concentrations over time in diaphragm tissue measured by microdialysis (**A**) and in serum measured by blood gas analysis (**B**) in CMV (closed circles) and STIM (solid circles) animals. Significant differences are given for the relation CMV vs. STIM. Microdialysis measures did not start until 4 hrs after the onset of anaesthesia for implantation of the probes (see methods for clarification). Broken line displays time point of microdialysis-start where animals were randomized into the interventional groups. Values are displayed as means ± standard error.

Diaphragmatic lactate concentrations increased in the CMV group transiently from 5 until 7 hrs after induction of CMV (Fig. 3) and these changes were significant (p < 0.001). Conversely, in the STIM group lactate concentrations dropped during this initial time period (maximal difference of 2.6 vs. 0.9 mmol L^−1^ after 5 hours and 20 minutes), but these changes were not significant. Diaphragmatic lactate concentrations changed significantly between the STIM group and CMV (p < 0.001) (Fig. 3A). This difference could not be observed in systemic serum lactate concentrations, which did not change significantly for both interventional groups and remained within normal ranges (Fig. 3B).

### CMV altered diaphragmatic pyruvate levels but not the lactate/pyruvate ratio

**Figure 4:**
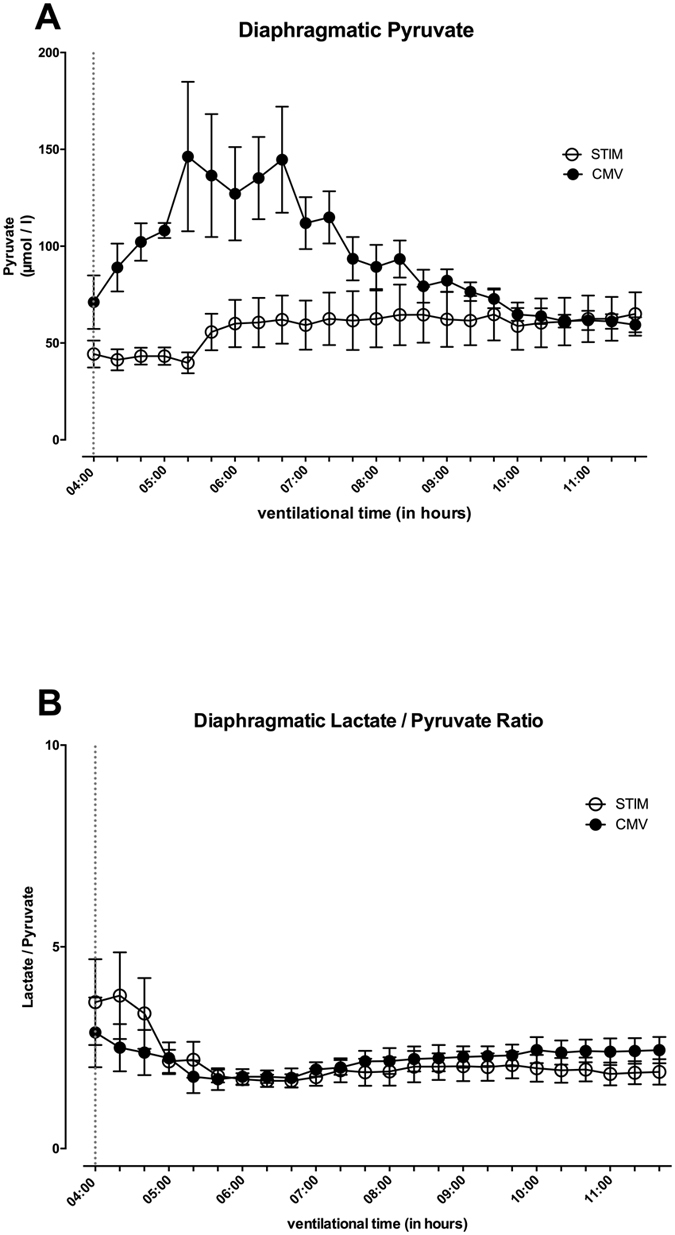
Pyruvate concentrations over time in diaphragm tissue measured by microdialysis (**A**) and lactate/pyruvate ratio (**B**) in CMV (closed circles) and STIM (solid circles) animals. Significant differences are given for the relation CMV vs. STIM. Broken line displays time point of microdialysis-start where animals were randomized into the interventional groups. Values are displayed as means ± standard error.

While diaphragmatic pyruvate concentrations in STIM remained stable over time, analogue pyruvate levels in CMV increased significantly with a maximal difference of 40 vs. 146 μmol L^−1^ after 5:20 hours between CMV and STIM, respectively; these changes between the groups were significant (p < 0.001). Afterwards levels of diaphragmatic pyruvate remained comparably stable in both groups (Fig. 4A). Significant differences in the lactate/pyruvate ratio were detected between STIM and CMV, but these changes may be due to the intersection of variables; the actual data demonstrate nearly equal changes during the experiment (Fig. 4B).

### Diaphragmatic glu**co**se levels were unaltered by CMV

**Figure 5:**
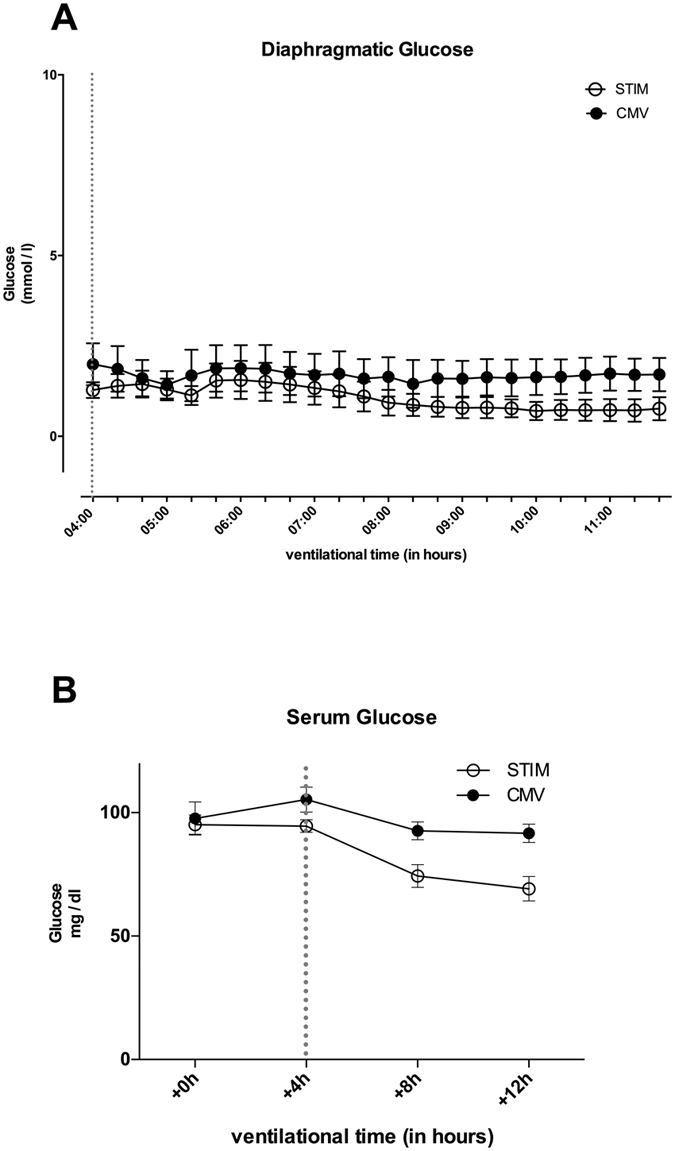
Glucose concentration over time in diaphragm tissue measured by microdialysis (**A**) and in serum measured by blood gas analysis (**B**) in CMV (closed circles) and STIM (solid circles) animals. There were no differences over time between groups. Broken line displays time point of microdialysis-start where animals were randomized into the interventional groups. Values are displayed as means ± standard error.

Diaphragmatic glucose concentrations were comparable in both groups with a temporal decline in STIM (maximal difference of 1.7 vs. 0.7 mmol L^−1^ after 11 hours) (Fig. 5A). There was no difference in serum glucose concentrations, which were stable for both interventional groups within normal ranges (Fig. 5B). The lactate/glucose ratio was unaltered and did not differ between both groups.

### 12 hrs of CMV did not lead to diaphragmatic atrophy

Dimensions of diaphragmatic muscle fibres, as assessed by ATPase assay, did not differ between both groups (data not reported).

### CMV induced proteolysis but did not effect lipid peroxidation

**Figure 6:**
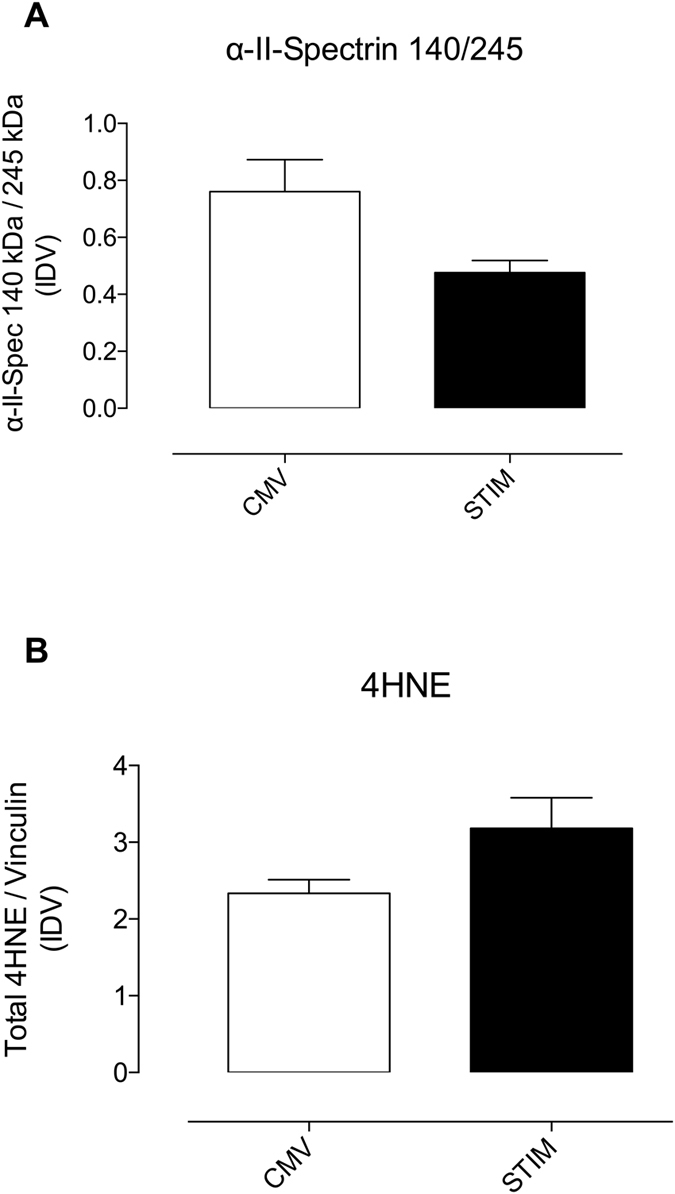
Panel A: Calpain-1 activity as marker of protein breakdown assessed as ratio of the uncleaved (245 kDa) to the 140 kDa breakdown product of α-II-spectrin. CMV = Controlled mechanical ventilation, STIM: Stimulated breathing by phrenic pacing. Panel B: Density of the 4- hydroxynonenal (4- HNE) bands (100, 60, 50, 37 kDa) was analysed as an indicator of lipid peroxidation, relative to the global amount of Vinculin detected via Western blot technique presented as integrated density value (IDV). CMV = Controlled mechanical ventilation, STIM: Stimulated breathing by phrenic pacing. Values are displayed as means ± standard error.

The levels of the VIDD-relevant protease calpain-1, assessed as ratio of the uncleaved (245 kDa) to the 145 kDa breakdown product of α-II-spectrin, were significantly increased in the CMV Group compared to STIM (Fig. 6, Panel A). The 120 kDa breakdown product as maker of caspase-3 activity was not significantly altered between the groups. 4-HNE as marker of lipid peroxidation was decreased in CMV compared to STIM, but did not reach statistical significance (Fig. 6, Panel B).

## Discussion

Our study confirms the feasibility of utilizing a novel technique of inserting regular pacer wires into the diaphragm to stimulate contractions and generate sufficient tidal volumes. The study also provided insight into the metabolic changes from the interstitial space of the inactive diaphragm during mechanical ventilation and an actively contracting diaphragm as discussed below.

Diaphragmatic pacing is a well-established technique to assist patients with high cervical paraplegia, by placing stimulators directly on the diaphragm surface. However, this is performed in a very small, predefined patient population. Thousands of patients undergo cardiac surgery every year and the majority can be successfully extubated. Several comorbidities may affect diaphragmatic function prior to mechanical ventilation; especially in COPD patients or in septic patients (caused by endocarditis f.e.) when the diaphragm is affected by proteolysis and impaired biomechanical properties. This ultimately depresses diaphragm function, adding to impairments during an ICU stay which is termed “ICU-acquired diaphragmatic weakness”^21^. Depending on comorbidities and preoperative status, some of these prior defined premorbid patients are ventilated for days and may require weeks for successful weaning^22^. The possibility to maintain an active diaphragm, even during deep sedation (which is often obligate during surgery), may mitigate or alleviate the weaning process, shorten ventilation time and therefore decrease associated costs, clinical time, and morbidity and mortality rates. Importantly, placing regular pacer electrodes in the surgical procedure during extracorporal circulation should not increase patient risk or lengthen the operation time significantly. However, the feasibility in human patients and the detection of patients at risk that might profit from artificial pacing, must be tested in further studies.

In two animals STIM animals, the stimulation voltage had to be increased over time to maintain a sufficient tidal volume. Changes in voltage may be indicative of oedema generation in the surrounding tissue, increasing current impedance. However, we did not find any histological abnormalities (i.e., no signs of trauma or burns) in the stimulated tissue, even if a higher voltage was required. The maximal voltage used for pacing was 12 Volts in one animal (Fig. 2), which is less than the maximum voltages used for epimyocardial pacing after surgery. The resulting current will be different due to the different resistance of epimyocardial and the perineural tissue of the parietal pleura, but we did not detect any influence on ECG activity or elicited arrhythmias. Importantly stimulation via pacer electrodes preserved the diaphragm from metabolic changes during ventilation in temporal periods of the investigated experimental period of 12 hrs CMV. However, we cannot conclude that such preserved diaphragm function would occur during an entire ventilation episode.

CMV caused a transient rise in both lactate and pyruvate concentrations in the diaphragm. Due to the nature of the microdialysis technique, we determined changes in the interstitial space. We did not detect changes in the lactate/pyruvate ratio as the directional change in both metabolites was the same.

During CMV diaphragmatic metabolism is disturbed in several ways. Firstly, generation of mitochondrial reactive oxygen species are major contributors to cellular apoptosis^23^. Secondly, there is a time-dependent reduction in diaphragm perfusion, leading to a potentially ischemic state after prolonged CMV^7^. Concomitant to this reduction in diaphragm perfusion with long-term CMV, there is also a reduction in basal oxygen uptake (VO_2_) within the diaphragm^7^. Therefore, unlike peripheral skeletal muscle that does not display hypoxia or a rapid reduction in blood flow and VO_2_ with disuse, the diaphragm rapidly alters its metabolic rate (lower VO_2_) with disuse, potentially as a result from the lowered perfusion. Indeed, we have observed an increased mRNA expression of HIF-1A in the diaphragm with prolonged CMV, suggesting a potential hypoxic microenvironment within the diaphragm^24^. The lactate/pyruvate ratio (LPR) has traditionally been used clinically to reflect potential tissue anaerobic metabolism. In our study, we did not see a change in the LPR over time, suggesting a sustained aerobic metabolism or a concomitant reduction in both diaphragm blood flow and metabolism over time. Recently, it has been demonstrated that the LPR may not independently indicate shifts in metabolism, and that lactate concentrations in the dialysate should also be interpreted^25^. We did observe a transitory lactate increase beginning after 5 hrs of CMV, suggesting a potential shift in metabolism. In previous studies we detected a decrease in blood flow to the diaphragm and a reduction of microvascular PO_2_ indicating that there is the possibility of tissue hypoxia in the diaphragm after 6 hrs of CMV^7^. Subsequent investigations in a model of 24 hrs of MV demonstrated an up-regulation of some hypoxia sensitive factors (i.e., HIF1-A)^24^. However, those previous studies were limited to discrete time points for data collection (i.e., no continuous measurements), which limited the ability to detect temporal changes in O_2_ delivery or tissue metabolism. In the current study, we collected data on tissue metabolism in 20 min intervals. Therefore, lactate changes (and possibly changes in diaphragm QO_2_) may be dynamic and not linearly decreasing over time.

Several reasons could explain these dynamic vs. linear changes in hypoxic regulation: The diaphragm is a muscle with a high blood flow reserve and can rapidly increase O_2_ delivery to match changes in VO_2_^26^. This blood flow reserve is greatly reduced after 6 hours of MV^7^. Nevertheless, although arteriole dynamics are regulated by many vasoactive factors (e.g., nitric oxide and metabolites) that are highly dependent on absence or presence of muscle contraction, supply of oxygen and other nutrients, and superoxide production fluctuations of these vasoactive signals may explain this transient nature of metabolic changes, although future studies addressing this are needed.

All animals were ventilated for 4 hours due to the need of the surgical implantation of microdialysis probes and stimulation electrodes. However, after this time point, despite similar baseline values of lactate and pyruvate in both groups, it was clear that activation of the diaphragm (in the STIM group) was sufficient to stop potential metabolic changes from occurring as in the CMV group over time. This supports the findings of Martin and colleagues who demonstrated that even short activation of the diaphragm during surgery can ameliorate mitochondrial disturbances that are existent even after short lasting MV^27^.

We did not observe atrophy of the diaphragm muscle in our experimental model. Based on the data of Jung and colleagues this could not be expected in a time period of several hours in pigs^28^. Interestingly, we can detect an increase of calpain-1 as one protease leading to diaphragmatic atrophy^29^. Due to the fact that neither the elevation of the breakdown product of caspase-3 as major protease in apoptotic and VIDD signalling^30^ nor 4-HNE as marker of oxidative stress^31^ reached significance we cannot profoundly state that proteolytic pathways are activated. Nevertheless, although we did not observe atrophy, we did detect early metabolic changes possibly originating the ROS generation, protease activation and apoptotic pathways that contribute to “VIDD”.

## Limitations 

Our study has several limitations. This study was performed in a pre-clinical porcine model for proof-of-concept. To enable translation to clinical care, we used cardiac pacer wires commonly used in the operating theatre and certified within the European Economic Area by CE marking for the use in patients. Only a limited time frame was investigated in this study which, when translated into a clinical scenario, would be too short to determine if these wires would stimulate sufficiently for weeks after insertion. Our data describing diaphragmatic metabolism could not be recorded directly after the induction of CMV due to the need of the appropriate surgical implementation. Therefore, it is unclear whether changes in diaphragm metabolism occurred in the initial 4 hrs of surgical preparation prior to our first microdialysis measurement, but this time period was necessary to ensure surgical accuracy. Furthermore, implantation of wires, chest tubes and microdialysis probes in awake spontaneously breathing pigs, as an ideal control is not possible due to the nature of the experimental setting.

Although our animals were deeply sedated we cannot exclude the possibility that some contractions of the diaphragm occurred during the study period. We chose to not use neuromuscular blocking agents to reduce potential interference with our drug cocktail^32^. Martin and colleagues demonstrated that intermittent contractions (i.e., duty cycle of ~1.7%) via phrenic nerve stimulation can attenuate mitochondrial dysfunction in mechanically ventilated, cardiac surgery patients^27^. Therefore, because we cannot assert that the diaphragm was completely inactive, such potential contractions may have interacted with the observed metabolic changes between STIM and CMV. We avoided the use of neuromuscular blocking agents to reduce interference with potentially influencing drugs; however, the dose of fentanyl used in our study (0.02 mg/kg) would also likely stop spontaneous breathing activity.

## Conclusions

We successfully implemented a novel method to stimulate the diaphragm by CE certified pacer wires. Using this technique we generated a sufficient tidal volume to preserve gas exchange despite deep sedation without any side effects for the 12 hrs time frame of measurements used herein. Furthermore, transient nutritional/metabolic changes in the diaphragm were observed during CMV, which might be connected to metabolic disturbances as one pathophysiologic origin of ventilator-induced diaphragmatic dysfunction. Importantly, re-activation of the diaphragm by field stimulation near the phrenic nerve (to mimic spontaneous breathing) was sufficient to protect the diaphragm from metabolic disturbances over time in the observed setting of 12 hrs of CMV, although it is unknown how such interventions would affect diaphragm function in longer ventilation episodes. This suggests that such a stimulation procedure may help mitigate ICU-related diaphragm dysfunction and minimize their need for CMV.

## Additional Information

**How to cite this article**: Breuer, T. *et al*. Kinetics of ventilation-induced changes in diaphragmatic metabolism by bilateral phrenic pacing in a piglet model. *Sci. Rep.* **6**, 35725; doi: 10.1038/srep35725 (2016).

## Supplementary Material

Supplementary Information
