# Supplementary material for: Kinetics of ventilation-induced changes in diaphragmatic metabolism by bilateral phrenic pacing in a piglet model
Source: Sci Rep. 2016 Oct 19;6:35725. doi: 10.1038/srep35725 (PMC5069624; doi:10.1038/srep35725)
Supplement: Supplementary Information [file srep35725-s1.pdf]

Kinetics of ventilation-induced changes in diaphragmatic metabolism by bilateral phrenic pacing in a piglet model

Thomas Breuer [1, 3*], Nima Hatam [2], Benjamin Grabiger [1], Gernot Marx [3], Bradley J Behnke [4], Joachim Weis [5], Ruedger Kopp [3], Ghislaine Gayan-Ramirez [6], Norbert Zoremba [1,7] and Christian S Bruells [3]

* Corresponding Author

[1] Department of Anaesthesiology, University Hospital of the RWTH Aachen, Aachen, Germany

[2] Department of Thoracic and Cardiovascular Surgery, University Hospital of the RWTH Aachen, Aachen, Germany

[3] Department of Intensive and Intermediate Care, University Hospital of the RWTH Aachen, Aachen, Germany

[4] Department of Kinesiology, Johnson Cancer Research Institute, Kansas State University, Manhattan, Kansas, USA

[5] Institute of Neuropathology, University Hospital of the RWTH Aachen, Aachen, Germany

[6] Laboratory of Pneumology, Katholieke Universiteit Leuven, Leuven, Belgium

[7] Department of Anaesthesiology, Sankt Elisabeth Hospital, Gütersloh, Germany

Supplemental Figure 1:

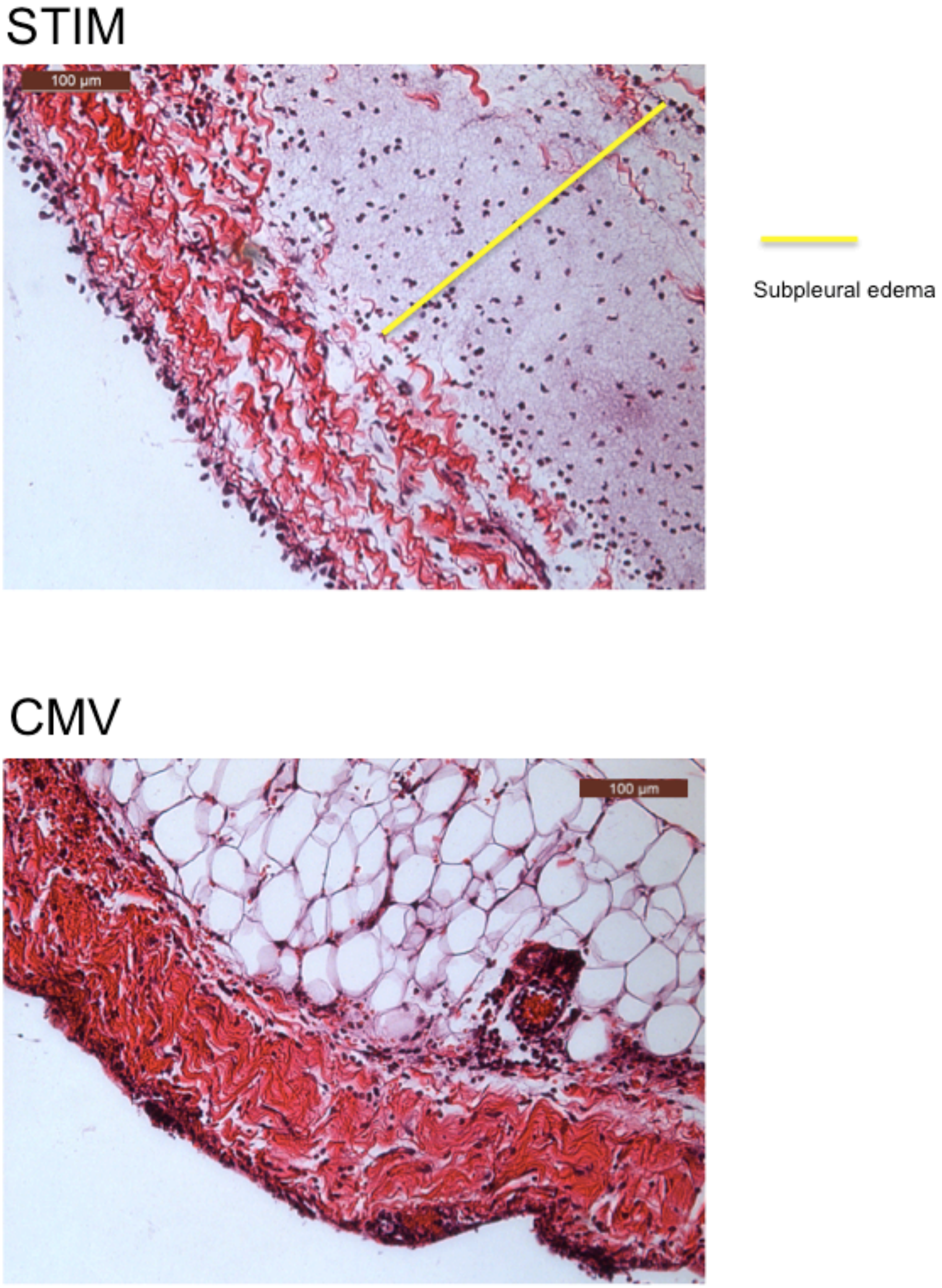

Supplemental Figure 1: Histological slices (hematoxylin-eosin staining, magnification of 10x) of insertion area of the stimulation electrodes in the pleural tissue. STIM animals showed a subpleural edema, marked as yellow line in the picture STIM. This was not found in CMV animals.

Supplemental Figure 2:

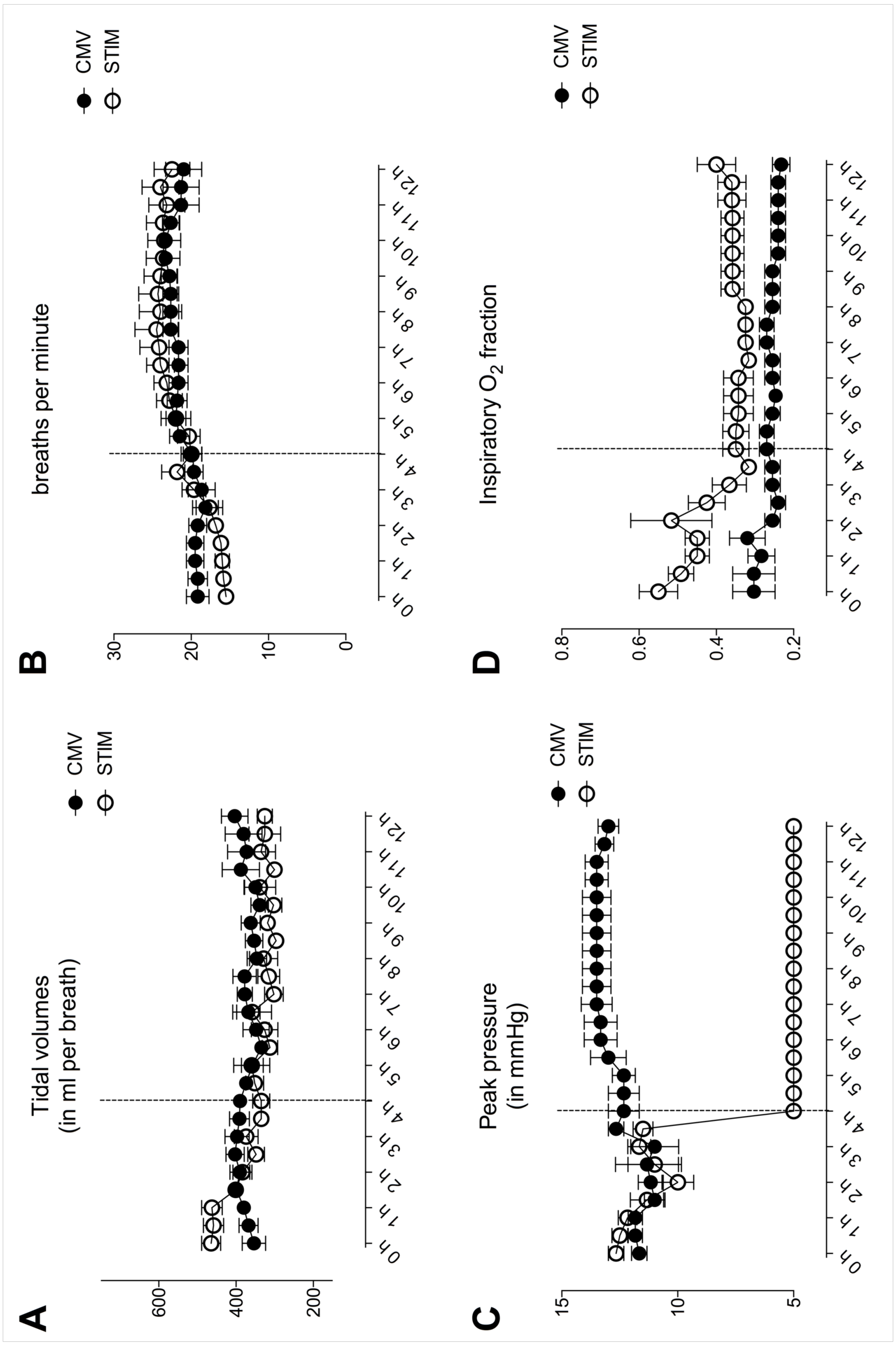

Supplemental Figure 2: Ventilator settings used in both groups. Tidal volumes expressed as ml per breath (Panel A). Respiratory frequency expressed as breaths per minute (Panel B). Peak airway pressure expressed in mmHg (Panel C). Inspiratory oxygen fraction displayed as percentage of total inspiratory gas concentration (Panel D). CMV (closed circles) and STIM (solid circles). Broken line displays time point of microdialysis-start where animals were randomized into the interventional groups. Values are displayed as means ± standard error.

Supplemental Figure 3:

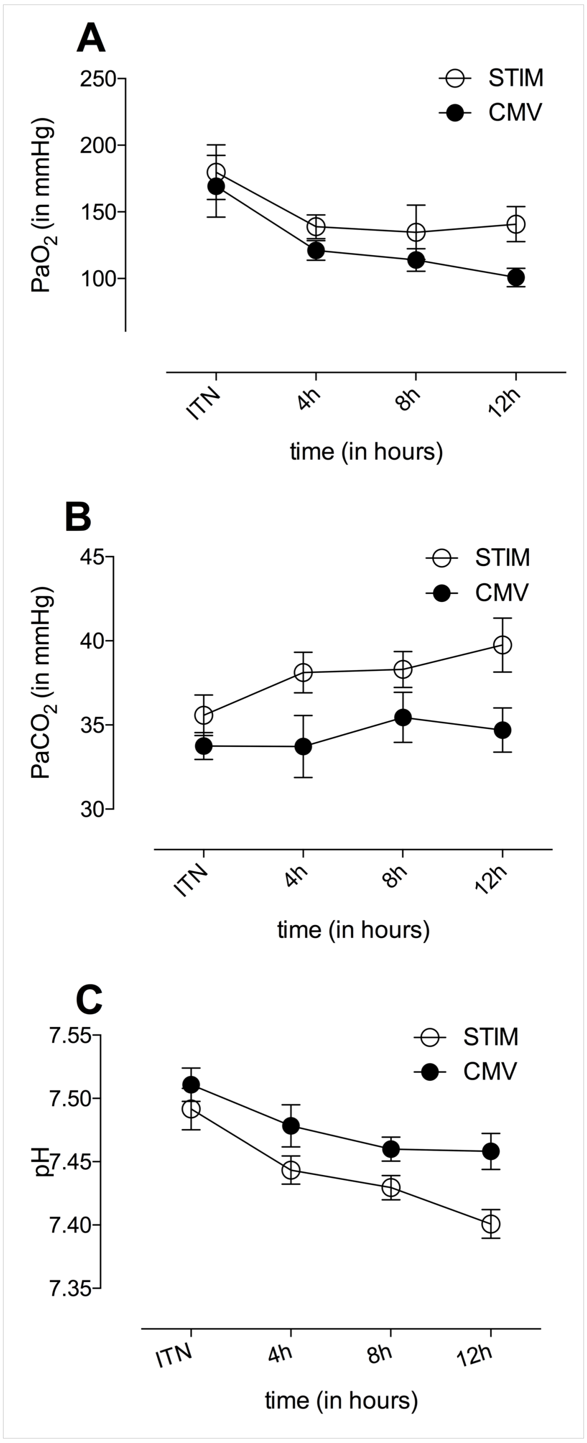

Supplemental Figure 3: Parameters of arterial blood gas analysis taken every 4 hours in both groups. Arterial partial pressure of oxygen expressed in mmHg (Panel A). Arterial partial pressure of carbon dioxide expressed in mmHg (Panel B). Arterial pH as potential of hydrogen (Panel C). CMV (closed circles) and STIM (solid circles). Values are displayed as means ± standard error.
